# Harnessing soft tissue sarcoma with low-dose pazopanib – a matter of blood levels

**DOI:** 10.1186/s12885-018-5043-9

**Published:** 2018-12-03

**Authors:** Stefanie L. Groenland, Daniela Katz, Alwin D. R. Huitema, Neeltje Steeghs

**Affiliations:** 1 Department of Medical Oncology and Clinical Pharmacology, The Netherlands Cancer Institute – Antoni van Leeuwenhoek, Amsterdam, The Netherlands; 2 Department of Oncology, Assaf Harofeh Medical Center, Zrifin, Israel; 3 Department of Clinical Pharmacy, University Medical Center, Utrecht University, Utrecht, The Netherlands; 4 Department of Pharmacy & Pharmacology, The Netherlands Cancer Institute – Antoni van Leeuwenhoek, Plesmanlaan 121, 1066 CX Amsterdam, The Netherlands

**Keywords:** Therapeutic drug monitoring, Pazopanib, Toxicity

## Abstract

**Background:**

Pazopanib is a tyrosine kinase inhibitor indicated for the treatment of renal cell carcinoma and soft tissue sarcoma. Despite the high inter-patient variability in pharmacokinetic exposure, pazopanib is administered at a fixed dose of 800 mg once daily (QD). Pharmacokinetic exposure is linked to both efficacy and toxicity. In this case report, we illustrate the value of therapeutic drug monitoring by describing two patients with adequate pazopanib trough concentrations (C_min_) at an eight times lower than standard dose.

**Case presentation:**

Patient A is a 69-year-old woman with metastatic leiomyosarcoma who had significant toxicities and a high C_min_ on the standard dose. While dose reductions to 200 mg QD and later 200 mg every other day were made, pazopanib C_min_ remained above the efficacy threshold. Patient B is a 50-year-old male with metastatic angiosarcoma and a history of Gilbert syndrome. Pazopanib treatment was initiated at the standard dose of 800 mg QD, but was reduced to 200 mg QD 1-week-on - 1-week-off due to total bilirubin elevation. Pazopanib C_min_ was adequate in this patient as well.

**Conclusion:**

It could be valuable to measure pazopanib levels in case of dose reductions due to toxicity, as exposure could still be adequate at considerably lower than standard doses.

## Background

Pazopanib is a tyrosine kinase inhibitor mainly targeting the vascular endothelial growth factor receptor and is indicated for the treatment of advanced renal cell carcinoma and soft tissue sarcoma [1]. Despite the high inter-patient variability in exposure (40–70%), pazopanib is administered at a fixed oral dose of 800 mg once daily (QD) [1, 2]. Suttle et al reported a clear exposure-response relationship, with patients with a pazopanib trough concentration (C_min_) above 20.5 mg/L having a significantly longer progression-free survival (PFS). Also, an exposure-toxicity relationship has been demonstrated, with an increasing incidence of toxicities such as hypertension, diarrhea, elevated alanine aminotransferase levels, stomatitis and hand-foot syndrome with increasing pazopanib plasma concentrations [3]. It has been shown that patients are unlikely to tolerate C_min_ ≥ 50 mg/L for a prolonged period of time [4]. In this case report, we illustrate the value of therapeutic drug monitoring (TDM) for pazopanib by describing two patients with pazopanib C_min_ above the efficacy threshold of 20.5 mg/L at an eight times lower than standard dose.

## Case presentation

### Case A

**Fig. 1:**
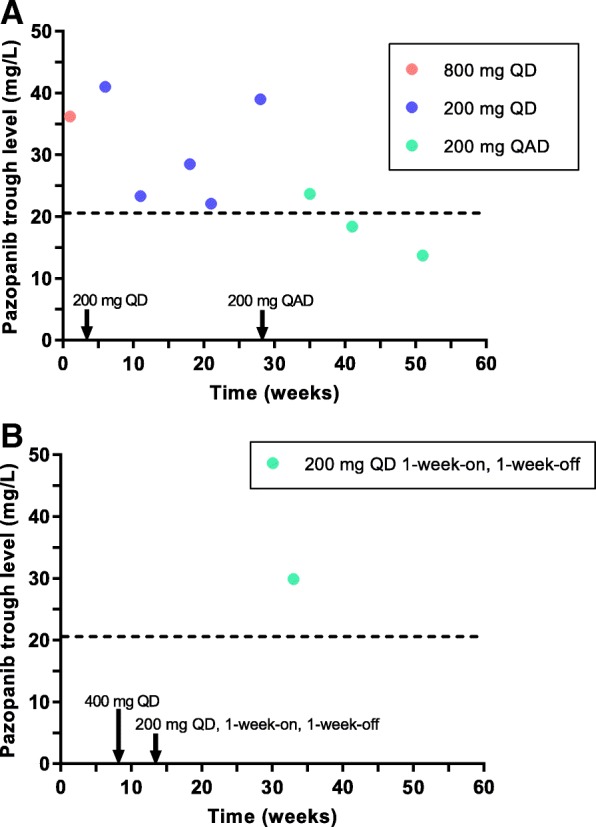
Calculated pazopanib trough levels. **a**: For patient A, pazopanib dose was rapidly reduced to 200 mg QD in the first three weeks and further reduced to 200 mg every other day in week 28. Corresponding doses of pazopanib were: week 0–1 800 mg QD, week 1–2 stop, week 2–2.5 600 mg QD, week 2.5–3 stop, week 3–11.5 200 mg QD, week 11.5–13 stop, week 13–28 200 mg QD, week 28–59 200 mg every other day. **b**: For patient B, pazopanib dose was reduced to 400 mg QD after two months and to 200 mg QD 1-week-on, 1-week-off after three months. Corresponding doses of pazopanib were: week 0–6 800 mg QD, week 6–8 stop, week 8–12 400 mg QD, week 12–13 stop, week 13–39 200 mg QD 1-week-on, 1-week-off. The dashed line indicates the pharmacokinetic target of C_min_ ≥ 20.5 mg/L. The arrows indicate the times at which doses were changed. C_min_ = minimum blood concentration, QAD = every other day, QD = once daily

We present a 69-year-old woman with a history of metastatic leiomyosarcoma, for which pazopanib treatment was initiated at the standard dose of 800 mg QD, after she progressed upon first-line chemotherapy with doxorubicin. During the first month of treatment pazopanib was temporarily withheld twice due to significant toxicities, including fatigue, nausea, vomiting and syncope. Pazopanib plasma concentrations were measured and C_min_ was calculated using the formula proposed by Wang et al [5], showing high pazopanib trough levels (36.1 mg/L and 41 mg/L). Pazopanib treatment was resumed after sequential dose reductions to 600 mg QD and 200 mg QD. The last dose was well tolerated despite mild liver enzyme disorders and hypertension. During the following months, the patient developed diarrhea and hypothyroidism, after which pazopanib was further reduced to 200 mg every other day. Pazopanib C_min_ remained adequate at this eight times lower than standard dose at first, although the last two measurements were below the efficacy-threshold (Fig. 1a). Unfortunately, 14 months after start of treatment, progressive disease was observed, after which chemotherapy with trabectedin was started.

### Case B

The second case is a 50-year-old male with metastatic angiosarcoma and a history of Gilbert syndrome, previously treated with 6 cycles of doxorubicin in combination with ifosfamide. Pazopanib treatment was started at the standard dose of 800 mg QD. Shortly hereafter, total bilirubin increased to twice the upper limit of normal with only minimal elevation of direct bilirubin, after which pazopanib was halted. Upon normalization of bilirubin, pazopanib treatment was resumed at a reduced dose of 400 mg QD and later 200 mg QD 1-week-on – 1-week-off. At the end of the on-treatment week pazopanib C_min_ was 29.9 mg/L (Fig. 1b). The patient is still on treatment now, nine months after pazopanib initiation, with a partial remission.

## Discussion and conclusion

It is remarkable that even these unusually low doses of pazopanib lead to an adequate exposure, defined by C_min_ ≥ 20.5 mg/L. [3] In the absence of TDM, when doses would be reduced according to the Summary of Product Characteristics (SPC), treatment would probably have been discontinued in these patients, as the SPC considers the exposure at 200 mg QD as markedly reduced and insufficient to obtain a clinically relevant effect [1]. However, we observed an adequate C_min_ at considerably lower than standard doses and treatment could have been continued for 14 and 9 months (ongoing response), respectively. This is a relative long duration of response taking into account the median PFS of 4.6 months for sarcoma patients found in the phase 3 trial [6]. Although the efficacy threshold of C_min_ ≥ 20.5 mg/L has been established in patients with renal cell carcinoma [3], a similar trend has been demonstrated in sarcoma patients [7].

Gilbert’s syndrome, uridine diphosphoglucuronate glucuronosyltransferase 1A1 (UGT1A1) polymorphism, has been associated with pazopanib-induced hyperbilirubinemia, which was probably the case in the second patient [8]. Given the benign etiology of this condition, continuing pazopanib treatment was possible, although in reduced dose.

With regard to obtaining the most adequate exposure, the dosing regimen of 200 mg every other day is most rational, as with the 200 mg 1-week-on, 1-week-off regimen patients will probably not have an adequate exposure in the off-treatment week. Unfortunately, we do not have any trough concentrations in the off-treatment week of patient B available.

As we illustrated with these two cases, it could be valuable to measure pazopanib levels in case of dose reductions due to toxicity, as C_min_ could still be above the efficacy threshold at considerably lower than standard doses.

## Abbreviations

C_min_: Minimum blood concentration
PFS: Progression-free survival
QAD: Every other day
QD: Once daily
SPC: Summary of product characteristics
TDM: Therapeutic drug monitoring
UGT1A1: Uridine diphosphoglucuronate glucuronosyltransferase 1A1
